# Sex and diet-dependent gene alterations in human and rat brains with a history of nicotine exposure

**DOI:** 10.3389/fpsyt.2023.1104563

**Published:** 2023-02-10

**Authors:** Javier Vargas-Medrano, Luis M. Carcoba, Guadalupe Vidal Martinez, Zuber D. Mulla, Victoria Diaz, Alejandra Ruiz-Velasco, Fabian Alvarez-Primo, Gabriela Colina, Sergio D. Iñiguez, Peter M. Thompson, Laura E. O’Dell, Bharathi S. Gadad

**Affiliations:** ^1^Department of Psychiatry, Texas Tech University Health Sciences Center, El Paso, TX, United States; ^2^Southwest Brain Bank, Texas Tech University Health Sciences Center, El Paso, TX, United States; ^3^Paul L. Foster School of Medicine, Texas Tech University Health Sciences Center, El Paso, TX, United States; ^4^Department of Psychology, The University of Texas at El Paso, El Paso, TX, United States

**Keywords:** nicotine, smokers, sex, diet, *CHRNA10*, *CERKL*, *SMYD1*, *FA2H*

## Abstract

**Introduction:**

Chronic nicotine exposure induces changes in the expression of key regulatory genes associated with metabolic function and neuronal alterations in the brain. Many bioregulatory genes have been associated with exposure to nicotine, but the modulating effects of sex and diet on gene expression in nicotine-exposed brains have been largely unexplored. Both humans and rodents display motivation for nicotine use and the emergence of withdrawal symptoms during abstinence. Research comparing pre-clinical models with human subjects provides an important opportunity to understand common biomarkers of the harmful effects of nicotine as well as information that may help guide the development of more effective interventions for nicotine cessation.

**Methods:**

Human postmortem dorsolateral prefrontal cortex (dLPFC) tissue BA9 was collected from female and male subjects, smokers and non-smokers (*N* = 12 per group). Rat frontal lobes were collected from female and male rats that received a regular diet (RD) or a high-fat diet (HFD) (*N* = 12 per group) for 14 days following implantation of a osmotic mini-pump (Alzet) that delivered nicotine continuously. Controls (control-s) received a sham surgical procedure. RNA was extracted from tissue from human and rat samples and reversed-transcribed to cDNA. Gene expression of *CHRNA10* (Cholinergic receptor nicotinic alpha 10), *CERKL* (Ceramide Kinase-Like), *SMYD1* (SET and MYD Domin Containing 1), and *FA2H* (Fatty Acid 2-Hydrolase) in humans was compared to rats in each subset of groups and quantified by qPCR methods. Additionally, protein expression of FA2H was analyzed by immunohistochemistry (IHC) in human dLPFC.

**Results:**

Humans with a history of smoking displayed decreased *CHRNA10* (*p* = 0.0005), *CERKL* (*p* ≤ 0.0001), and *SMYD1* (*p* = 0.0005) expression and increased *FA2H* (*p* = 0.0097) expression compared to non-smokers (*p* < 0.05). Similar patterns of results were observed in nicotine exposed vs. control rats. Interestingly, sex-related differences in gene expression for *CERKL* and *FA2H* were observed. In addition, ANCOVA analysis showed a significant effect of nicotine in a sex-different manner, including an increase in *CERKL* in male and female rats with RD or HFD. In rats exposed to an HFD, *FA2H* gene expression was lower in nicotine-treated rats compared to RD rats treated with nicotine. Protein expression of *FA2H* (*p* = 0.001) by IHC was significantly higher in smokers compared to non-smokers.

**Conclusion:**

These results suggest that a history of long-term nicotine exposure in humans alters the expression of sphingolipid metabolism-related (*CERKL*, *SMYD1*, and *FA2H*) and neuronal (*CHRNA10*) marker genes similarly as compared to rats. Sex- and diet-dependent differences appear in nicotine-exposed rats, critical in regulating sphingolipid metabolism and nicotinic acetylcholine receptors. This research enhances the construct validity of rat models of nicotine usage by showing a similar pattern of changes in gene expression in human subjects with a smoking history.

## 1. Introduction

According to the National Institute on Drug Abuse (NIDA), an estimated 23.6 million Americans over the age of 12 display nicotine dependence (1). Tobacco use continues to be the leading cause of mortality in the United States, with cigarette smoking killing an average of 480,000 Americans yearly (2). In 2020, the economic burden of cigarette smoking cost in the United States was estimated to be $864 billion (3). Nicotine is the primary reinforcing component of tobacco and e-cigarettes, and its motivational effects are due to the activation of nicotinic acetylcholine receptors (nAChRs) in the brain (4). Recently, it has been recognized that several nAChRs sub-unit genes play a role in modulating nicotine dependence. The gene *CHRNA10* encodes the α10 subunit of the nAChR. For example, the *CHRNA10* gene and its variants appear to modulate the trajectory of nicotine dependence in humans (5). After initial exposure to nicotine, tobacco, and e-cigarette, users often need to increase their use and avoid the physical and negative affective states that emerge during withdrawal. Thus, experiencing the pleasurable effects of nicotine and avoiding withdrawal symptoms that contribute to maintained use and relapse behavior (4). Long-term nicotine use leads to gene expression alterations that promote the development of the trajectory of nicotine dependence (6). Chronic nicotine exposure also induces epigenetic modifications, including DNA methylation of several genes in different areas of the brain, such as in the medial prefrontal cortex (mPFC), dorsolateral prefrontal cortex (dLPFC), orbitofrontal cortex (OFC), and nucleus accumbens (NAc) (7). Anatomical evidence supports the view that the rat dorsolateral prefrontal cortex is related to human dLPFC (8, 9). Functionally the dLPFC is responsible for the maintenance and regulation of impulsivity, decision-making, cognitive control of mood and other executive functions (10). A recent study by Onos et al. showed that rats encode actions and outcomes, using over delays, body posture or distinctions in running path, and these aspects are tracked by dorsal and medial PFC neurons (11). Combining these elements of the rat and human dLPFC might be compared at a fundamental level.

Smoking induces DNA methylation and changes in gene expression in the brain and widespread alterations in pulmonary, cardiovascular, hepatic, and adipose tissue (12). Several studies have demonstrated that DNA methylation can be used as a predictor in human behavior for nicotine dependence, withdrawal, or relapse (6). Numerous factors affect epigenetic modifications in human smokers, including the duration of nicotine exposure, brain region, sex, and diet (6). Chronic nicotine exposure may also alter the expression of a variety of other nicotinic acetylcholine receptors (nAChR)-interacting genes, proteins, and micro RNA (miRNA) in a sex-dependent manner (6). Withdrawal symptoms are experienced in a sex-dependent manner; women experience greater negative affective states during withdrawal from chronic use as compared to men (13, 14), consistent with some rodent studies (15, 16). The existing treatments for alleviating withdrawal are less effective in women vs. men (14). Chronic smoking behavior is the primary cause of cancer and pulmonary disease, and a combination of a high-fat diet (HFD) drastically increases the chances of developing cardiovascular diseases and diabetes (17, 18). Obesity linked to high-fat diet is considered to be one of the major risk factors for cognitive dysfunction in the brain (19). Nicotine-containing products are often used for weight control (18). However, evidence from animal studies have shown that mice exposed to nicotine do not lose weight if they are exposed to nicotine and fed with HFD (18). Ingestion of a HFD regimen increases adipose tissue and leads to obesity. Adipose tissue has been associated with the secretion of pro-inflammatory cytokines, which induce inflammation that targets different peripheral organs, including the heart and brain (12, 20). A recent report from the O’Dell laboratory and others demonstrated that the magnitude of nicotine intake and withdrawal severity is greater in female rats compared to male rats that received a HFD vs. RD (21–23). These two risk factors could potentially lead to cognitive deficits in the brain. O’Dell laboratory demonstrated that the magnitude of nicotine intake and withdrawal severity is greater in female rats that received an HFD vs. RD, and this difference was greater compared to male rats (21–24). The modulating effects of sex and diet on gene expression in nicotine-exposed brains in humans or rats requires further investigation. Therefore, validating rodent models used in pre-clinical studies that assess gene expression alteration due to nicotine exposure is critical. In addition, they may lead to discoveries that will inform the development of more effective and specialized treatment strategies for nicotine cessation in men and women.

To achieve this goal, the present study investigated the deregulatory effects of sex on gene expression in the brains of postmortem humans with a history of smoking and in rodents exposed to nicotine. Also we investigated the deregulatory effects on gene expression of two different diets in the brains of rodents exposed to nicotine. In our rat study the use of high-fat diet (HFD) represents the unhealthy diet that obese people may consume in their regular life. To determine the genes of interest, we used preliminary data from a whole transcriptome sequencing in frontal lobe of postmortem human brain of subjects with major depressive disorder, associated with smoking behavior. The selected genes *CHRNA10*, *SMYD1*, *CERKL*, and *FA2H* were significantly dysregulated compared to other genes. In the present study, we studied the expression of these genes in the human postmortem subjects, to confirm a construct validity of rat models exposed to nicotine to that of human brain (Supplementary Figure 1). Then, molecular and statistical methods were combined to cross-validate changes in gene expression in the brains of postmortem humans and rats exposed to nicotine. Finally, we hypothesized that within the frontal lobes, there would be a similar pattern of sex- and diet-dependent changes in gene expression of metabolic and neuronal markers following chronic exposure to nicotine.

## 2. Materials and methods

### 2.1. Human subjects with a history of smoking

Human postmortem brain tissue from dorsolateral prefrontal cortex (dLPFC) Broadmann area-9 (BA9), subjects with a history of smoking and controls without a history of smoking (*N* = 12 per group) were used for this study. All demographic and clinical characteristics of human subjects are described in Table 1A. The age range was between 38 and 79 years old; the postmortem intervals (PMI) range was between 23 and 29 h. The RNA integrity number (RIN) value was 7 ± 1 for controls and 6 ± 2 for smokers., while the brain pH values of 6 ± 0.3 for controls and 6 ± 0.5 for smokers, which did not differ significantly across either group (see Table 1A). The samples were obtained from the Southwest Brain Bank (SWBB), Department of Psychiatry, Texas Tech University Health Sciences Center-El Paso (TTUHSC-EP), with prior consent from the next-of-kin (NOK). The SWBB collection of postmortem tissue for research is conducted under the jurisdiction of the State of Texas Anatomical Review Board and the TTUHSC-EP Institutional Review Board, which regulates the NOK consent and interviews (IRB# E16046), consistent with our previously published work (25). Subjects with a major neurological disease, such as Alzheimer’s or Parkinson’s disease or brain tumors, were excluded from the study. Intact fresh brains were transported to the SWBB, and the cerebrum was hemisected, then each hemisphere was cut consecutively into 1-cm thick coronal serial blocks. After performing the cuts, brain sections were frozen in 2-methylbutane (Fisher-Scientific, Cat# O3551-4) and stored at −80°C. A board-certified neuropathologist analyzed all tissue samples, free of any confounding gross and/or microscopic neuropathology. For this study, the prefrontal cortex corresponding to BA9 was dissected from the left hemisphere.

**TABLE 1:** Demographics for postmortem human subjects (A) and rat frontal lobes with regular diet (B), and high-fat diet (C).

(A) Human postmortem frontal lobes		
	Non-Smokers	Smokers
*N*	12	12
Mean death age ± SD (years)	72 ± 7	52 ± 14
Median death age	72	53
% Nicotine	0	100%
Male/Female	8/4	6/6
PMI (hours) ± SD	23 ± 8	29 ± 12
Brain pH ± SD	6 ± 0.3	6 ± 0.5
RIN ± SD	7 ± 1	6 ± 2
**(B) Frontal lobe from regular diet rats**		
	**Control-s**	**Nicotine**
*N*	12	12
% Nicotine	0	100%
Male/Female	6/6	6/6
RIN ± SD	7.9 ± 1	7.8 ± 1
**(C) Frontal lobe from high-fat diet rats**		
	**Control-s**	**Nicotine**
*N*	12	11
% Nicotine	0	100%
Male/Female	6/6	5/6
RIN ± SD	8.1 ± 1	8.5 ± 1

### 2.2. Exposure to nicotine in rats

Male and female Wistar rats were acquired from an outbred stock from Envigo, Co (Indianapolis, IN, USA). The rats were pair-housed under humidity- and temperature-controlled conditions under a reverse light cycle (8 a.m. lights off) with food and water *ad libitum*. Adult rats between 60 and 75 days of age were anesthetized with isoflurane (1-3%) and implanted with an osmotic mini-pump (model 2ML2; 5.0 μL/h; Durect Corporation, Inc., Cupertino, CA, USA) that continuously delivered either saline-controls (control-s) or nicotine for 14 days (3.2 mg/kg/day; base), controls received sham surgery. Our nicotine formulation was (−) nicotine hydrogen tartrate salt that was obtained from NIDA Drug Supply Program (Research Triangle, Bethesda, MD, USA). This nicotine dose has been shown to produce similar levels in female and male rats (26). Following surgery, rats received topical antibiotic ointment (Neosporin^®^) on the wound and subcutaneous administration of the analgesic flunixin (2.5 mg/kg; Vedco, St Joseph, MO, USA). Animals were then divided into groups that consumed regular diet (RD) or high-fat diet (HFD). The HFD consisted of 5.1 kcal/g, 60% of total calories from fat, and the HFD was purchased from Envigo (Cat# TD06414, Madison, WI, USA). The diet was stored at 4°C, and was replenished on daily basis. Control rat as received standard RD chow from the same vendor that consisted of 3.1 kcal/g, 17% kcal of total calories from fat. The HFD regimen was given during the 14 days of nicotine exposure to assess the potential additive effects of this diet manipulation on gene changes produced by nicotine. The HFD regimen was not intended to induce an obesity-related phenotype. Each diet group had a control-s (*N* = 12) or nicotine-treated (*N* = 12) condition with six males and six females per group (see Table 1B). After 14 days of control-s or nicotine exposure, rats were sacrificed using isoflurane and rapid decapitation. Prefrontal cortex samples were collected and frozen on dry ice. All procedures were approved by the Institutional Animal Care and Use Committee of The University of Texas at El Paso (IRBNet ID # 1559961-8).

### 2.3. RNA extraction from tissues

For the postmortem human brain samples, approximately ∼100 mg of frozen BA9 frontal lobe (*N* = 12 non-smokers and *N* = 12 smokers, see Table 1A) were removed and homogenized in 1 mL of QIAzol lysis buffer reagent (Qiagen, Germantown, MD, USA) using Polytron PT 2100 with a tip PT-DA 07/2EC-D100 (Fisher Scientific, Waltham, MA, USA) in a 4°C cold room. From the homogenate, RNA was isolated using RNeasy Lipid Tissue Mini Kit (Cat# 74804, Qiagen) accompanied by on-column DNase digestion with RNase-Free DNase Set (Cat# 79254, Qiagen), as per manufacturer’s instructions. RNA integrity was assessed by electrophoresis (Agilent 2200 TapeStation system, Santa Clara, CA, USA), and only RNA samples with RIN values of ≥6 were subjected to further analyses, as shown in Table 1A. Finally, cDNA was synthesized using a High-Capacity RNA-to-cDNA kit (Thermo-Fisher Scientific, 4388950) according to the manufacturer’s protocol. RIN values are shown in Table 1A.

For the rat brain samples, approximately ∼100 mg of the frozen frontal lobe was collected from all groups of rats and homogenized in 1 mL of QIAzol lysis buffer reagent (Qiagen, Germantown, MD, USA) using a Bullet Blender homogenizer from Next Advance Inc. (Troy, NY, USA) in a 4°C cold room. Samples were homogenized for 1 min at speed 10. Homogenate was used to isolate RNA by RNeasy Lipid Tissue Mini Kit (Cat# 74804, Qiagen) accompanied by on-column DNase digestion with RNase-Free DNase Set (Cat# 79254, Qiagen), as per the manufacturer instructions (27). RNA integrity was assessed by electrophoresis (Agilent 2200 TapeStation system, Santa Clara, CA, USA), and only RNA samples with RIN values of ≥6 were included for further analyses. cDNA was synthesized using a High-Capacity RNA-to-cDNA kit (Thermo-Fisher Scientific, 4388950) according to the manufacturer’s protocol. RIN values are shown in Table 1B, C.

### 2.4. Quantitative polymerase chain reaction (qPCR)

Sequences of mRNA were acquired from the Nucleotide Database at the National Center for Biotechnology Information; primers were designed using the Primer3 server (28). Primers were synthesized using a commercial service (Millipore-Aldrich, St. Louis, MO, USA), and sequences of each primer are shown in Supplementary Table 1 for humans and rats. Quantitative PCR (qPCR) reactions were carried out using PowerUP SYBR Green Master Mix (Cat# A25777, Thermo-Fisher Scientific, Waltham, MA, USA). Relative gene expression analysis was performed by a qPCR measured in ViiA 7 Real-Time PCR system (Thermo-Fisher Scientific) with QuantStudio Real-Time PCR System 1.3 (Thermo-Fisher Scientific). Ct values for each human gene were normalized using the Ct values of two housekeeping genes, *GAPDH* and *RPL30*. Ct values for each rat gene were normalized to the Ct values of one housekeeping gene, *GAPDH*.

### 2.5. Immunohistochemistry

A fresh postmortem tissue from the human brain dLPFC block was dissected and immediately fixed in 10% neutral buffered formalin. The tissue was shipped to STRL histology/immunochemistry laboratory at the University of Texas Health Science Center San Antonio, where the tissue was processed and embedded into paraffin blocks. The embedded paraffin blocks were sectioned into 10 microns in thickness using a microtome RM2125 RTS (Leica, Deer Park, IL, USA). The sections obtained were then mounted onto a glass superfrost plus gold slides (Fisher Scientific, Hampton, NH, USA) and allowed to adhere overnight. The paraffin was removed from the tissue and rehydrated with a sequence of xylene and ethanol washes, outlined with a hydrophobic pen, and then washed twice with a wash buffer containing PBS and 0.025% triton X-100 before blocking it with blocking medium containing PBS, 10% FBS, and 1% BSA for 1 h at room temperature. After the blocking step, the tissue was rewashed with the wash buffer. Then the tissue was stained with the an appropriate antibody specific to neurons (NeuN)-Alexa Fluor 488 (Cat# 54761S, Cell Signaling Technology, Danvers, MA, USA), and fatty acid 2-hydroxylase (FA2H)-Alexa Fluor 594 (Cat# NBP2-72192AF594, Novus Biologicals, Centennial, CO, USA) overnight and protected from light at 4°C. Finally, the slides were washed twice with wash buffer. Cell nuclei were stained with DAPI (Cat# 4083S, Cell Signaling Technology Inc., Danvers, MA, USA), and mounted with Fluromount-G slide mounting medium (Cat# OB100-01, Fisher Scientific). At least 3 images per tissue sample (subject) were randomly acquired among dLPFC sections on an LSM 700 confocal microscope (Zeiss, Oberkochen, Germany), for each image 3 filters channels of green (Alexa fluor 488), red (Alexa fluor 594) and blue (DAPI) were acquired. All fluorescent images were captured using a Zen 2009 software with the same brightness and contrast for all the images. Images acquired were analyzed using the software ZEN 3.1 Histo tool, to determine the arithmetic mean intensity of each channel. The arithmetic mean intensity for each sample and channel was averaged and plotted to a histogram using Prism 6 Software (GraphPad Inc., San Diego, CA, USA) and data was analyzed by a *t*-test. In addition, Zen 2009 software was used to generate a height map of a representative section image of the tissue showing the fluorescence intensity in the Z-direction.

### 2.6. Statistical analysis

Three-way analysis of variance (ANOVA) models were fit using The GLM Procedure in SAS 9.4 (SAS Institute, Inc., Cary, NC, USA). The dependent variable was the fold change in gene expression. A separate ANOVA model was fit for each of the four genes. The following binary factors were entered in each model: treatment, sex, and diet. For each gene, a three-way interaction (Treatment × Sex × Diet) and all possible two-way interactions were tested for statistical significance using an alpha of 0.05. If the *p*-value for an interaction term was greater than 0.05, then it was dropped from the model. Marginal (least square means) were calculated and pairwise differences were tested for statistical significance. The Tukey–Kramer method was used to adjust for multiple comparisons. For MANOVA and ANCOVA we used diet, treatment, sex, as binary factors in the model for each genes. Student *t*-test was performed by Prism 6 Software (GraphPad Inc., San Diego, CA, USA). For histograms, data represent the mean ± SEM of *n* = 12 for humans and *n* = ∼12 for rats. Multi-Way ANOVA, Tukey’s for *post hoc* (*p* < 0.05), ANCOVA (*p* < 0.05), and Interaction plots were generated *via* base R through RStudio (RStudio, Boston, MA, USA) after processing the data with the “dplyr” (29) R-package, and assessing for normality through a Shapiro Wilks test. Further, Heatmaps were produced with R package “heatmaply” (30) displaying a matrix of varying gene expression fold changes from the two significant genes (*CERKL* and *FA2H*) in three different groups along a color gradient ranging from red (higher expression) to black (lower expression).

## 3. Results

### 3.1. Nicotine-dependent alterations in gene expression

**FIGURE 1:**
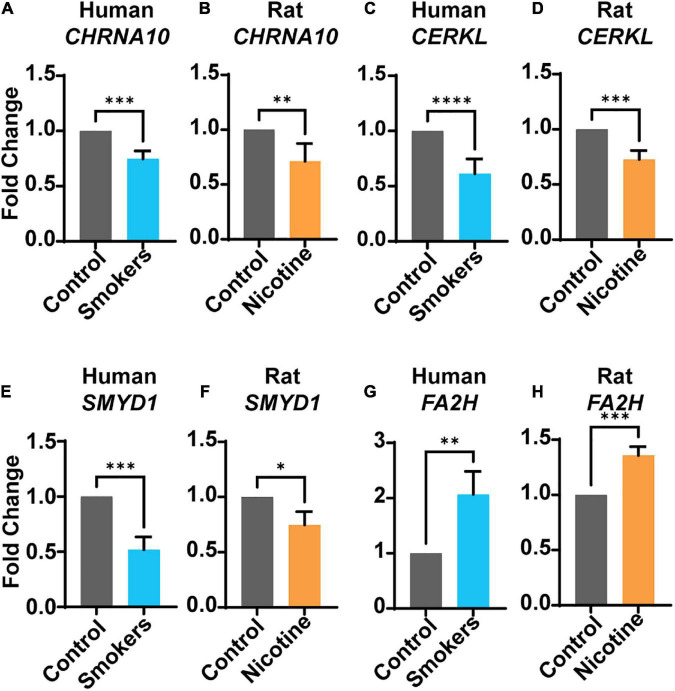
Gene expression levels in human smokers or rats treated with nicotine. Human (blue bars) smokers or rats (orange bars) treated to nicotine were analyzed. The following genes: *CHRNA10* **(A,B)**, *CERKL* **(C,D)**, and *SMYD1* **(E,F)**, were all significantly under-expressed in both species. Gene *FA2H* **(G,H)** was significantly higher in both species. Each bar represent the mean ± SEM of 12 humans or rats per group; *p < 0.05; ^**^p < 0.01; ^***^p < 0.001, ^****^p < 0.0001 (*t*-test).

Gene expression levels were analyzed in human and rat brain tissues for the genes of interest, *CHRNA10*, *CERKL*, *SMYD1*, and *FA2H.* In humans, the genes *CHRNA10* (*p* = 0.0005) (Figure 1A), *CERKL* (*p* = < 0.0001) (Figure 1C), and *SMYD1* (*p* = 0.0005) (Figure 1E) were significantly lower in human smokers (blue bars) compared to control (non-smokers, gray bars). Moreover, the gene expression of *FA2H* (Figure 1G) (*p* = 0.009) was significantly higher compared to the control group. In the rat model, the gene expression of *CHRNA10* (*p* = 0.004) (Figure 1B), *CERKL* (Figure 1D) (*p* = 0.0005), and *SMYD1* (Figure 1F) (*p* = 0.023) were significantly lower in rats exposed to nicotine (orange bars) compared to the control-s (gray bars). In addition, *FA2H* (Figure 1H) (*p* = 0.0005) was significantly higher than the control-s; the same trend was observed in humans.

### 3.2. Sex-dependent alterations in gene expression

**FIGURE 2:**
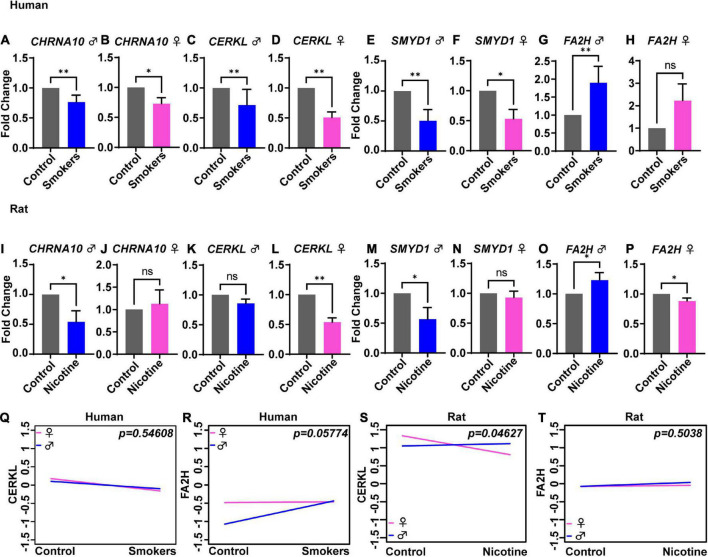
Sex-dependent changes in gene expression levels in human smokers and rats treated with nicotine. Gene expression expressed in fold change was analyzed in males (blue) and females (pink) for humans **(A–H)** and rats **(I–P)**. ANCOVA analysis showed no significant differences between human males and females for the following genes: *CERKL* **(Q)** and *FA2H* **(R)**. In rats, ANCOVA analysis showed significant differences between male and female rats for gene *CERKL* **(S)**, but not for *FA2H* **(T)**. Each bar represents the mean ± SEM of six humans or rats; ns, no significant; *p < 0.05; ^**^p < 0.01 (*t*-test).

To evaluate sex differences in humans, the gene expression fold change values were split by sex and then graphed and analyzed separately. The genes *CHRNA10* [Figure 2A (*p* = 0.003) and Figure 2B (*p* = 0.047)], *CERKL* [Figure 2C (*p* = 0.003) and Figure 2D (*p* = 0.001)], and *SMYD1* [Figure 2E (*p* = 0.003) and Figure 2F (*p* = 0.047)] were significantly lower in males and females, thus supporting no sex-dependent differences. However, *FA2H* [Figure 2G (*p* = 0.003) and Figure 2H (*p* = 0.158)] were significantly higher in males but not in females. An ANCOVA analysis was performed to further analyze the sex differences in gene expression. Expression of the *CERKL* gene showed no significant difference between male vs. female smokers (Figure 2Q) (*p* = 0.546). There were also no sex differences in the expression of *CHRNA10* and *SMYD1* (data not shown). ANCOVA analysis also revealed no significant difference for *FA2H* in male vs. female smokers. However, there was a trend for significant differences (*p* = 0.057) (Figure 2R).

In rats, the genes *CHRNA10* [Figure 2I (*p* = *0.015*) and Figure 2J (*p* = 0.318)] and *SMYD1* [Figure 2M (*p* = 0.0259) and Figure 2N (*p* = 0.3182)] were significantly lowered compared to control-s, but only in male rats and not in female rats. The gene *CERKL* [Figure 2K (*p* = 0.060) and Figure 2L (*p* = 0.002)] was lower only in female rats but not in male rats. Also, *FA2H* [Figure 2O (*p* = 0.0498)] expression was higher in male rats. Interestingly, in female rats (Figure 2P) (*p* = 0.015), *FA2H* was significantly lower than the effects observed in males, supporting a sex-dependent alteration. The ANCOVA analysis showed sex differences in the expression of the gene *CERKL* (Figure 2S) (*p* = 0.046), but not for *CHRNA10* (data not shown), *SMYD1* (data not shown), nor *FA2H* (Figure 2T) (*p* = 0.503).

### 3.3. Diet-dependent alterations in gene expression

**FIGURE 3:**
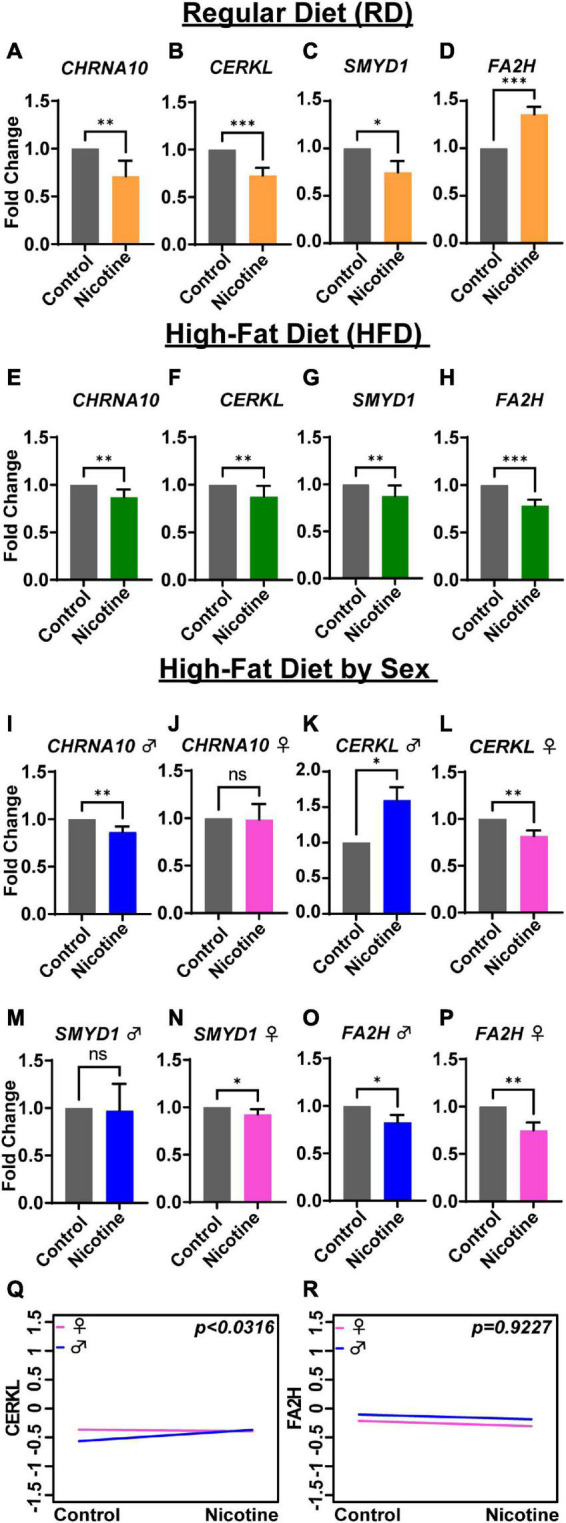
Diet- and sex-depending differences in gene expression levels in rats. Gene expression by fold change is shown for rats under a regular diet (yellow bars) **(A–D)**, high-fat diet (green bars) **(E–H)**, and high-fat by sex: male (blue bar) and female (pink bar) **(I–P)**. ANCOVA analysis showed sex differences between males and females for the *CERKL* gene **(Q)**, but not for *FA2H* **(R)**. Each bar represents the mean ± SEM of six rats; ns, no significant; *p < 0.05; ^**^p < 0.01, ^***^p < 0.001 (*t*-test).

In the rat tissues, changes in gene expression were compared following nicotine exposure in RD (Figures 3A–D) and HFD rats (Figures 3E–H). Gene expression of *CHRNA10* (*p* = 0.004), *CERKL* (*p* = 0.0005), and *SMYD1* (*p* = 0.023) (Figures 3A–C) was significantly lower in RD rats that were exposed to nicotine as compared to RD control-s. In contrast, *FA2H* (*p* = 0.0005) was significantly higher in RD rats exposed to nicotine (Figure 3D) compared to control-s. In addition, HFD rats that were treated with nicotine displayed significantly lower gene expression of *CHRNA10* (*p* = 0.006), *CERKL* (*p* = 0.0025), and SMYD1 (*p* = 0.002), similarly to RD control-s. Interestingly, expression of *FA2H* (Figure 3H) (*p* = 0.0002) in HFD rats treated with nicotine was significantly lower on an opposite trend as compared to RD rats that were exposed to nicotine (Figure 3D).

To discern sex differences in gene expression based on diet history, fold change values grouped into sex and analyzed. *CHRNA10* expression was significantly lower only in male rats (Figure 3I) (*p* = 0.004), but not significant in female rats (Figure 3J) (*p* = 0.3182). *CERKL* was significantly higher than control-s in male rats (Figure 3K) (*p* = 0.015) and significantly lower in female rats (Figure 3L) (*p* = 0.0022). As shown in Figure 3M, *SMYD1* was not significantly altered in male rats (*p* = 0.060) but was significantly lower in female rats (Figure 3N) (*p* = 0.0152) in a diet-dependent manner. On the other hand, *FA2H* was significantly lower in both male and female rats relative to their respective control-s (Figure 3O) (*p* = 0.0152) and (Figure 3P) (*p* = 0.0022), which was different from RD nicotine-treated rats (Figure 3D). Differences in gene expression based on sex were analyzed by ANCOVA. The analysis showed sex differences were significant only for the gene *CERKL* (Figure 3Q) (*p* < 0.0316), but not for *CHRNA10* and *SMYD1* (data not shown) or *FA2H* (Figure 3R) (*p* = 0.922).

### 3.4. Gene expression changes based on diet, sex, and treatment in rats

For *CHRNA10*, the three-way interaction term (Treatment × Sex × Diet) and the three two-way interaction terms (Treatment × Sex, Treatment × Diet, and Diet × Sex) were not statistically significant and hence were not retained in the ANOVA model. The p-value from the *F* test of the overall significance of the final model was 0.374. For each of the three factors, statistically significant differences in the marginal means were not detected (Table 2A). Table 2B presents the results of the ANOVA when fold change in the expression of *CERKL* was the outcome. The three-way interaction term (Treatment × Sex × Diet) was not statistically significant and therefore was dropped from the model. The Treatment × Sex interaction term was statistically significant. The *p*-value from the *F* test of the overall significance of the final model was <0.0001. The marginal mean fold changes in control-s (untreated) female rats and nicotine-treated female rats were 0.887 and 0.541, respectively (*p* = 0.011). None of the other treatment-sex pairwise tests of differences (Table 2B). The marginal mean fold change was higher in rats fed a RD than in rats who were fed a HFD: 1.076 vs. 0.395 (*p* < 0.0001). Results of the ANOVA in which fold change in the expression of *SMYD1* was the outcome are found in Table 2C. The three-way and two-way interaction terms were not statistically significant and hence were not retained in the ANOVA model. The *p*-value from the *F* test of the overall significance of the final model was 0.662. Statistically significant differences in the marginal means were not detected. ANOVA results for fold change in the expression of *FA2H* are reported in Table 2D. The three-way and two-way interaction terms were not statistically significant and hence were not retained in the ANOVA model. The *p*-value from the *F* test of the overall significance of the final model was 0.0003. The marginal mean fold change was 0.888 in nicotine treated rats and 0.734 in the untreated rats (*p* = 0.027). Rats with HFD had a lower marginal mean fold change than rats fed a RD: 0.669 vs. 0.953 (*p* = 0.0001).

**TABLE 2:** Results of the three-way analysis of variance (ANOVA) with treatment, sex, and diet (regular diet and high-fat diet) of fold change in the expression of (A) *CHRNA10* (*N* = 46), (B) *CERKL* (*N* = 47), (C) *SMYD1* (*N* = 47), (D) *FA2H* (*N* = 47).

Factor	Marginal mean	95% confidence limits	*P*-value*
**(A) *CHRNA10***			
**Treatment**			
Nicotine	0.992	0.766, 1.217	0.502
Control (Saline)	1.096	0.881, 1.311	
**Sex**			
Female	1.173	0.958, 1.388	0.102
Male	0.915	0.689, 1.141	
**Diet**			
High-fat diet	1.052	0.826, 1.278	0.918
Regular diet	1.036	0.821, 1.251	
**(B) *CERKL***			
**Treatment and sex**			
Nicotine, Female	0.541	0.391, 0.692	(See below)
Nicotine, Male	0.777	0.620, 0.934	
Control-s, Female	0.887	0.736, 1.037	
Control-s, Male	0.738	0.587, 0.888	
**Pairwise comparisons^†^: Treatment-by-sex interaction**			
Nicotine treated female vs. nicotine treated male	–	–	0.145
Nicotine treated female vs. control-s female	–	–	0.011
Nicotine treated female vs. control-s male	–	–	0.260
Nicotine treated male vs. control-s female	–	–	0.738
Nicotine treated male vs. control-s male	–	–	0.983
Control-s female vs. control-s male	–	–	0.496
**Diet**			
High-fat diet	0.395	0.287, 0.504	<0.0001
Regular diet	1.076	0.970, 1.182	
**(C) *SMYD1***			
**Treatment**			
Nicotine	0.709	0.572, 0.845	0.505
Control-s	0.772	0.639, 0.906	
**Sex**			
Female	0.780	0.647, 0.914	0.406
Male	0.701	0.564, 0.837	
**Diet**			
High-fat diet	0.707	0.570, 0.843	0.478
Regular diet	0.774	0.641, 0.908	
**(D) *FA2H***			
**Treatment**			
Nicotine	0.888	0.791, 0.984	0.027
Control-s	0.734	0.640, 0.829	
**Sex**			
Female	0.826	0.731, 0.921	0.653
Male	0.796	0.699, 0.892	
**Diet**			
High-fat diet	0.669	0.572, 0.765	0.0001
Regular diet	0.953	0.858, 1.05	

*Test of the null hypothesis that the two marginal means are equal in the population.

^†^*P*-values for the pairwise comparisons are adjusted for multiple comparisons using the Tukey–Kramer method.

### 3.5. Heatmap analysis of sex differences

**FIGURE 4:**
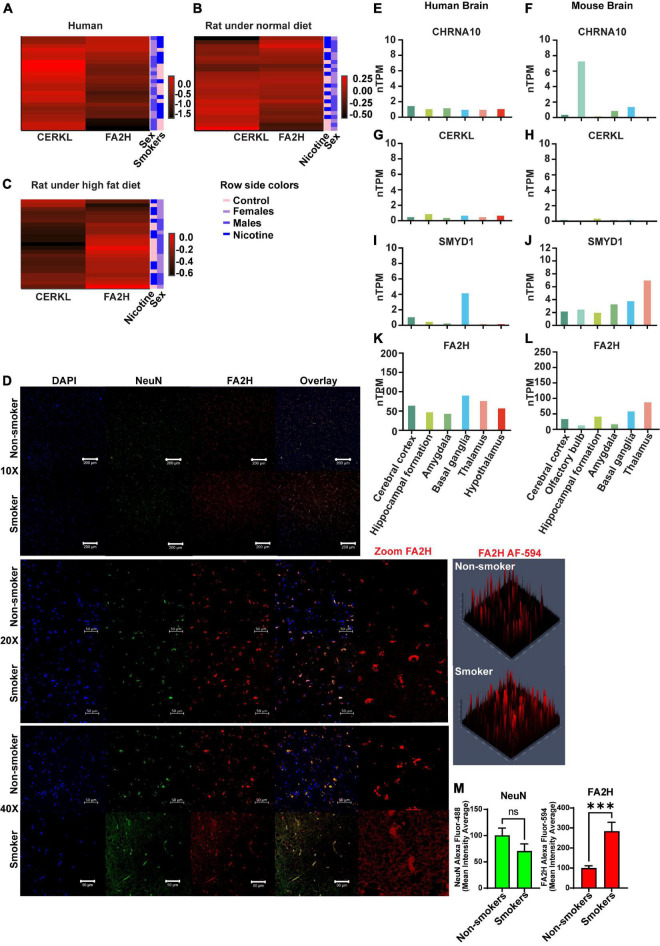
Heatmap analysis and immunohistrochemistry (IHC) of *FA2H* in human brains. Heatmap represents gene expression changes of *CERKL* or *FA2H* genes in humans **(A)** or rats that received an RD **(B)** or an HFD **(C)**. In panel **(D)**, a representative image of protein expression of FA2H (red color) was analyzed by IHC and confocal microscopy. Basal levels of *CHRNA10* **(E,F)** (https://www.proteinatlas.org/ENSG00000129749-CHRNA10/brain), *CERKL* **(G,H)** (https://www.proteinatlas.org/ENSG00000188452-CERKL/brain), *SMYD1* **(I,J)** (https://www.proteinatlas.org/ENSG00000115593-SMYD1/brain), and *FA2H* **(K,L)** (https://www.proteinatlas.org/ENSG00000103089-FA2H/brain) in normalized levels of RNA (nTPM) are shown in human or mouse brain (Courtesy of Human Protein Atlas proteinatlas.org). Histograms of the quantitative analysis of the IHC for NeuN or FA2H are shown **(M)**. Each bar represents the mean ± SEM of 3 images per subject, ns, no significant; ***p < 0.001 (*t*-test).

Heatmaps were generated to represent changes in gene expression in a visually comprehensive manner. The changes in gene expression are shown for humans (Figure 4A) and rats with RD (Figure 4B) or HFD (Figure 4C) conditions. Fold change values were assessed for normality prior to the matrix analysis. Images were processed in R-Studio with package “heatmaply” (30) displaying a matrix of varying gene expression in fold changes values from the two genes (*CERKL* and *FA2H*) having significant differences across sex and smoking history in humans or nicotine exposure in rats. The three different groups are displayed along a color gradient ranging from red (higher expression) to black (lower expression) in each panel. In human smokers, *FA2H* gene expression was higher relative to controls. In males, we observed higher expression; however, females have a lower expression than controls (Figure 4A).

*CERKL* gene expression in male rats that were fed with RD and were treated with nicotine had no discernible changes in gene expression; however, female rats displayed the opposite pattern of expression following nicotine exposure (Figure 4B). On the other hand, male rats treated with nicotine displayed higher expression of *FA2H*. Still, female rats did not display any appreciable changes in the heatmap (Figure 4B). HFD females (Figure 4C) showed no change in *CERKL* expression when exposed to nicotine, as evident by the relatively homogenous color distribution, in agreement with the interaction plots shown previously. For *FA2H* expression, HFD rats treated with nicotine displayed lower expression in females vs. males.

### 3.6. Expression of FA2H in human brains

We evaluated the FA2H protein expression in fresh-fixed paraffin-embedded tissue sections from human dLPFC, frontal lobes in smokers vs. non-smokers. The expression of FA2H (red) was considerably higher in smokers vs. non-smokers, as shown in Figure 4D with low (10X or 20X) or high (40X) magnification, which supported our findings by qPCR that was higher in smokers compared to non-smokers. The expression of NeuN protein, a specific neuronal marker, was used to stain neurons in green. Quantification of the fluorescence signal as mean intensity average was determined and plotted in a histogram (Figure 4M), showing the signal for FA2H was significantly higher (*p* = 0.001) in smokers vs. non-smokers.

Intrinsic expression of *CHRNA10*, *CERKL*, *SMYD1*, and *FA2H* in both human and mouse brains was obtained from the publically available datasets of Human Protein Atlas, which reveals that the genes *CERKL*, *CHRNA10*, *SMYD1*, and *FA2H* are expressed in both human and mouse brains (31)^1^. It also shows the expression in different areas (Figures 4E–L).

## 4. Discussion

This present study demonstrates an important integration of pre-clinical and human tissues to find common transcriptional gene signatures affected by chronic exposure to nicotine. To our knowledge, this is the first cross-validation of gene expression in postmortem human brains with a history of tobacco use and rats exposed to nicotine. Results of this study demonstrate a similar pattern of changes in genes *CHRNA10*, *CERKL*, *SMYD1*, and *FA2H* in postmortem humans and rats after nicotine exposure. In humans, the genes *CHRNA10*, *CERKL*, and *SMYD1* were significantly lower in both males and females following nicotine exposure relative to their respective controls (Figures 2A–F). On the contrary, gene expression of *FA2H* was higher only in male smokers (Figure 2G). Additionally, In rats, the expression of *CHRNA10*, *CERKL*, *SYMD1*, and *FA2H* were up-or down-regulated in a sex-dependent manner following nicotine exposure (Figures 2I–P). However, ANCOVA analysis only revealed significant sex differences for *CERKL* in rats receiving an RD (Figure 2S) and HFD (Figure 3Q).

Approximately 80% of smokers who want to quit relapse, and only <5% can maintain full abstinence (32). The prevalence of smoking in men has decreased in recent years in developed countries compared to women suggesting that women continue to use nicotine at higher rates than men (13). Multiple studies have shown sex-related differences in nicotine dependence and withdrawal in humans and rodents. In pre-clinical work in our laboratory, female rats have been shown to display greater negative affective states of withdrawal than males (26). However, there are reports that indicate heightened withdrawal symptoms in male vs. female rats (33). Women and men have biological differences; thus, the pharmacokinetics of drugs are different between them (14, 34). For example, DA transmission is altered with fluctuations in female-hormone levels across their menstrual cycle, an effect that is age-dependent (14). In this study the average age of female smokers was 53 years old which corresponds to the menopause cycle. This is not comparable with the age of the rats which were adults (60–73 days) but not in menopause state (which corresponds to 15–20 months) (35). This may explain some of the discrepancies in the results observed in this study. Preclinical studies have found sex-dependent differences in DA levels in NAc. For example, female rats display larger decreases in NAc DA levels during nicotine withdrawal compared to males. This sex difference in DA level during withdrawal may contribute to the larger expression of the negative affective states experienced by females during nicotine withdrawal (34).

Women tend to display higher rates of depressive behavior, which is influenced by fluctuations in hormone levels across the menstrual and reproductive cycle. In addition, anxiety and depression have been linked to high rates of smoking behavior and relapse rates (13). Studies in mice have shown that nicotine withdrawal produce anxiety-like behavior in animals and produces reactive oxygen species (ROS) in NAc, disrupting ROS homeostasis (32, 36). Therefore, high anxiety levels during smoking abstinence is believed to be one of the most significant factors driving high relapse rates in women (32).

In our study, *CHRNA10* displayed less expression in male and female smokers (Figures 2A, B) and even in male rats exposed to nicotine and fed RD (Figure 2I) or HFD (Figure 3I). The nicotinic acetyl cholinergic receptor (nAChR) subunit genes are part of a complex system unique for different areas of the brain in which genes are altered in a specific pattern following nicotine exposure (37). The nAChRs subunits are normally therapeutic targets for nicotine withdrawal (32). Preclinical and imaging *in vivo* studies have shown that the β2-nAChR subunit is significantly higher in male smokers in different parts of the brain in contrast with female smokers and controls who do not exhibit differences in this subunit availability (34). Nicotinic antagonists with a high affinity for nAChR have been used as therapeutic resources for smoking cessation (14). In our study, the gene encoding for the α10-nAChR subunit was consistently lower in women and men. More studies will be required to investigate its potential role as the therapeutic target, as has been done for other nAChR subunits. Nicotine activates different pathways that can disrupt the normal metabolic state in mice and humans (38). This is something to consider when looking for a therapeutic solution for smoking cessation.

The gene *CERKL* showed a significant lower expression in male and female smokers (Figures 2C, D) and female rats treated with nicotine with RD (Figure 2L) and HFD (Figure 3L). This finding may be related to the fact that women are more vulnerable than men to the negative health consequences associated with long-term nicotine use (39). *CERKL* is highly expressed in the cerebellum relative to other regions of the brain. Its function is related to vesicular trafficking ions and calcium channels (40). This gene is abundant in the lungs and kidneys as well (41). Huang et al., suggested that over-expression of *CERKL* protects the brain from ischemia-inducing mitophagy (42). At the cellular level, smoking can stimulate the production of ROS. Excess ROS can affect mitochondria, which are abundant in neuronal axons. Mitophagy is a process that will maintain homeostasis under these conditions. Several studies have suggested that *CERKL* is involved in cellular response against oxidative stress, and it is attached to mitochondrial membranes (41). Reviewing this data indicates that the protective effect of *CERKL* may be lost when the brain is chronically exposed to nicotine.

Our data showed that gene *SMYD1* was lower in expression in female and male smokers (Figures 2E, F), in male rats with RD (Figure 2M), and female rats that received the HFD regimen (Figure 3N). The *SMYD1* gene has been largely studied in muscle cells, but little is known about its function in other tissues, such as the brain (43). Its expression has been related to muscle formation, inflammation, and cancer (43, 44). Warren et al. study strongly suggest an active role of *SMYD1* in the regulation of mitochondrial energetics (45). The *SMYD1* gene belongs to a family of SMYD proteins related to immunity and inflammatory control and has been involved in neurodegenerative diseases (46).

Interestingly, the *SMYD1* gene has a histone methyl-transferase function involved in immune response, and epigenetic mechanisms can regulate gene expression (44). This may be related to the fact that smoking produces epigenetic modifications in DNA (7) and it did not change.

Further, the present study reveals that the expression of *FA2H* was significantly higher in male smokers (Figure 2G) and in nicotine-treated male rats (RD) (Figure 2O), and lower in female rats (Figure 2P). Interestingly, the gene *FA2H* displayed an opposite expression pattern (lower expression) in male and females rats with HFD (Figures 3O, P). Although gene expression data provided strong evidence with the history of tobacco use, we validated by performing immunohistochemistry on human dLPFC samples with a selective antibody to FA2H (red signal) protein (Figure 4D) and quantified expression of the FA2H. The confocal microscopy images showed a significant increase in expression/signal for FA2H (*p* = 0.001) in smokers compared to a non-smokers (Figure 4M).

*FA2H* is another gene of interest that is sex-related to male humans and rats exposed to nicotine. The expression of *FA2H* is abundant in the brain and colon tissue (47, 48). *FA2H* gene expression is very controversial; this gene is essential for myelin formation, present in myelin-forming cells, which are important for the transmission of electrical impulses and cognition (49, 50).

Additionally, down-regulation or up-regulation of *FA2H* has been associated with different types of cancer (47). Our study shows that when male rats are exposed to nicotine and fed with a HFD, *FA2H* is under-expressed (Figure 3H). *FA2H* is suggested to be used as a biomarker and a therapeutic target for breast and ovarian cancer (51, 52). In adenocarcinomas, *FA2H* is highly expressed (53). Some studies have shown that over-expression of *FA2H* may be related to a protective effect against cancer *via* stimulation of anti-cancer cellular mechanisms (54). Interestingly, when rats were fed with HFD, this gene was significantly under-expressed relative to control-s, presenting more questions about the role of *FA2H* in brain tissue (Figure 3H). Ingestion of a HFD is important when planning therapeutic strategies for smokers, especially if they are obese.

Smoking in combination with HFD exacerbates the risk for other diseases. For example, preclinical studies in male mice exposed to nicotine and fed with HFD resulted in increased production of ROS and mitochondrial abnormalities in muscles (55). Obese people may smoke to lose weight, which may be initially effective; however, after chronic exposure to nicotine, the nAChRs are desensitized, and nicotine has no effect on weight loss. Preclinical studies have demonstrated that mice with HFD maintain or even increase their body weight compared to controls fed with a RD (56). In addition, adipose tissue in obese people secrete cytokines and other inflammatory molecules leading to over production of ROS. During chronic exposure to nicotine and high ingestion of a HFD, there is an increase in lipolysis, leading to higher lipid distribution from adipose tissue to other organs (38). Also, there is a general cognitive deterioration in smoking people who also suffer from non-alcoholic fatty-liver disease, diabetes, and insulin resistance, among others (57). In addition, preclinical studies have shown that second–hand cigarette smoke may contribute to white matter degeneration, and it is considered a risk factor for diseases like AD (57). There is solid evidence that *CERKL*, *SMYD1*, and *FA2H* are implicated in the sphingolipids metabolism, either directly or indirectly. It will be interesting to understand how its regulation is affecting sphingolipids metabolism and their different pathways (58–60). Although we found some concerning in the literature regarding the expression of *CHRNA10*, *CERKL*, *SMYD1*, and *FA2H*, in the brain, the expression of these genes in different areas of the human and mouse brain were reported, including in the cerebral cortex (Figures 4E–L).

The interpretation of our results is in the context of nicotine dependence given that our regimen of nicotine exposure produces physical and negative affective states during withdrawal from 14 days of nicotine exposure (61). The connection to nicotine dependence is also based on the human literature showing that long-term nicotine use produces a withdrawal syndrome that is believed to promote continued nicotine use and relapse behavior (62). Current cessation approaches typically involve the reduction of withdrawal symptoms with nicotine replacement and/or pharmacological interventions that reduce withdrawal severity (63). However, we recognize that the changes in gene expression observed in the present study may also be related to other actions of nicotine, including changes in inflammatory, appetite, and/or vascular processes (64). Accordingly, nicotine exposure also produces changes in cell proliferation, oxidative stress, apoptosis, and DNA mutations that may have also contributed to the observed changes in our gene library examined here.

This study has some limitations to consider. For example, the age of postmortem tissue collection in human controls was lower than that of smokers. Postmortem samples were selected to ensure a history of chronic smoking, leading to a wider range of ages in this group. The rats were selected to represent the adulthood in humans. Rats representing the menopause cycle in female humans needed to be aged for 15–20 months. This practice is not suitable in lab animals in which resources are limited. Unfortunately, there was no information about the diet of our human donors, limiting our ability to directly assess diet in the human postmortem tissue compared to the HFD rats.

**FIGURE 5:**
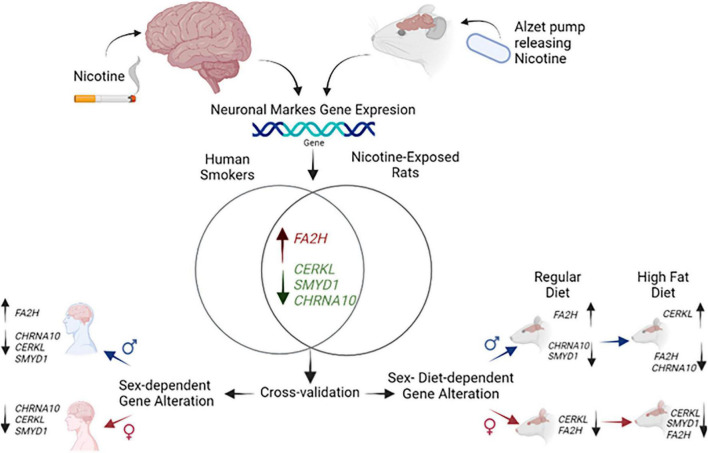
Model indicating gene dysregulation in nicotine-exposed humans and rats by sex and diet. Gene expression of *CHRNA10*, *CERKL*, *SMYD1*, and *FA2H* exposed to nicotine based on sex and diet.

To summarize, the present report provides novel information regarding changes in *CHRNA10*, *SYMD1*, *CERKL*, and *FA2H* that are expressed in a sex- and diet-dependent manner, and we are summarizing our study and findings in Figure 5. This work provides an operational framework for understanding the biological consequences produced by nicotine exposure and possibly identify novel targets for treatments that can help reduce the use of nicotine. This is the first study to provide cross-species validation within the common pattern of changes in genes found in humans and rats that also appear to be sex- and diet-dependent. Future studies are required to further understand the dysregulation of the genes *CHRNA10*, *CERKL*, *SMYD1*, and *FA2H*, to develop specialized interventions to reduce nicotine use and the harm produced by this drug in different vulnerable populations.

## Data availability statement

The datasets presented in this study can be found in online repositories. The names of the repository/repositories and accession number(s) can be found in the article/Supplementary material.

## Ethics statement

The studies involving human participants were reviewed and approved by Texas Tech University Health Sciences Center at El Paso. The patients/participants provided their written informed consent to participate in this study. The animal study was reviewed and approved by The University of Texas at El Paso.

## Author contributions

JV-M, GV, VD, AR-V, GC, LC, LO’D, PT, and BG worked in the collection and dissection of human postmortem brains, planned the experimental design, conducted the experiments (qPCRs and IHCs), drafted, edited, and finalized the manuscript. LC and LO’D performed the experiments in rat brains. ZDM assisted in biostatistics of the data, edited, and finalized the manuscript. FA-P and SDI assisted in manuscript edits. All authors reviewed and approved the final version of the manuscript.

## Footnotes

1
https://www.proteinatlas.org

## Supplementary material

Supplementary Figure 1 KEGG pathways for regulated genes in tobacco use disorders, the table shows the list of genes that are dysregulated and the top hits based on the fold change. Measurement on the x-axis represents −log10 (*p*-value).

Supplementary Table 1 Primers for *CHRNA10*, *CERKL*, *FA2H*, and *SMYD1* in humans and rats.
