# Supplementary figures and images for: Sex and diet-dependent gene alterations in human and rat brains with a history of nicotine exposure
Source: Front Psychiatry. 2023 Feb 10;14:1104563. doi: 10.3389/fpsyt.2023.1104563 (PMC9950561; doi:10.3389/fpsyt.2023.1104563)

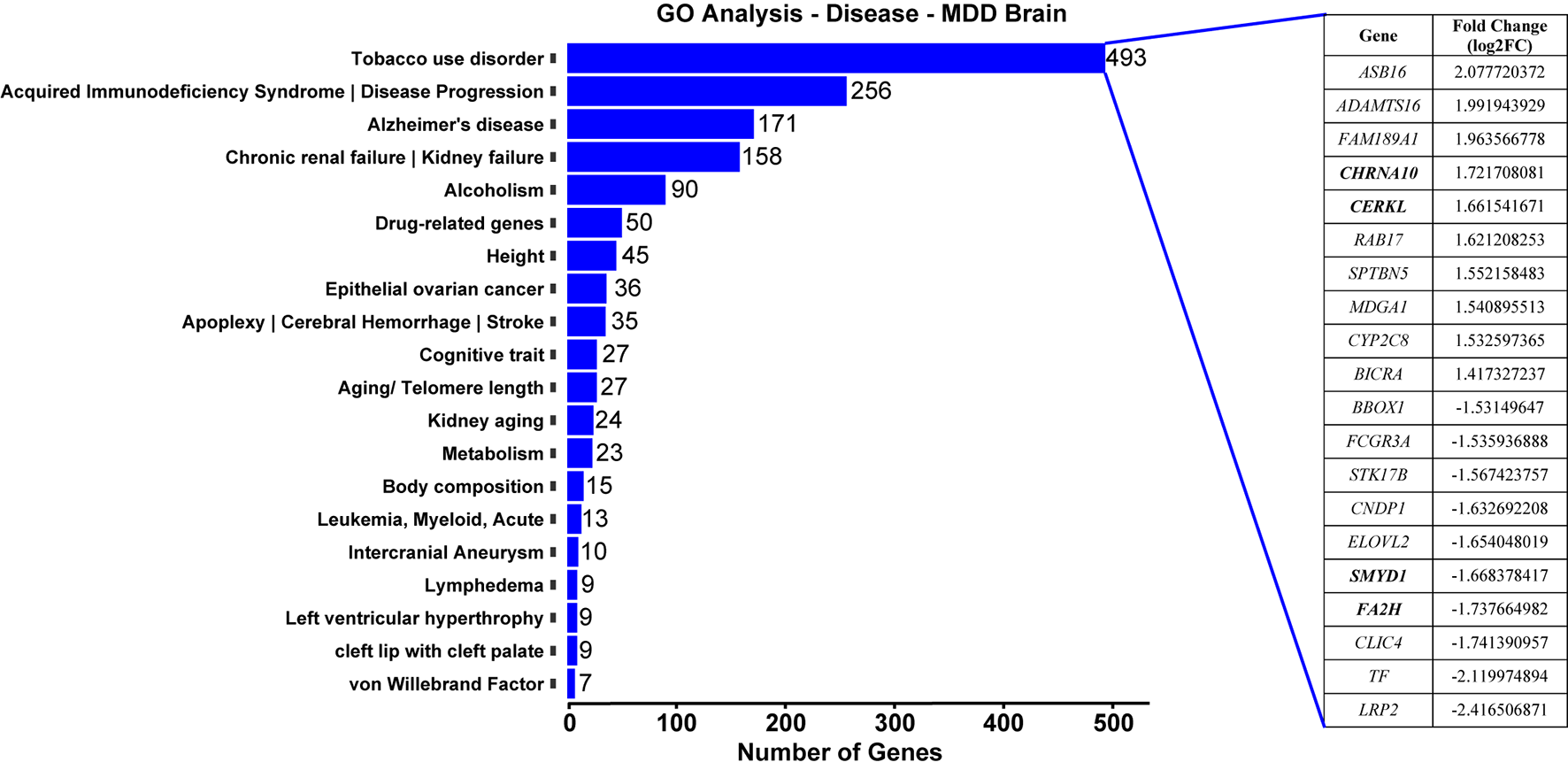

Supplement: Supplementary Figure 1 — KEGG pathways for regulated genes in tobacco use disorders, the table shows the list of genes that are dysregulated and the top hits based on the fold change. Measurement on the x-axis represents −log10 (p-value). [file Image_1.tif]
