# Supplementary material for: Sex and diet-dependent gene alterations in human and rat brains with a history of nicotine exposure
Source: Front Psychiatry. 2023 Feb 10;14:1104563. doi: 10.3389/fpsyt.2023.1104563 (PMC9950561; doi:10.3389/fpsyt.2023.1104563)
Supplement: Supplementary Table 1 — Primers for CHRNA10, CERKL, FA2H, and SMYD1 in humans and rats. [file Data_Sheet_1.docx]

**Supplementary Table 1: Primers for CHRNA10, CERKL, FA2H and SMYD1 in humans and rats.**

| Table A. Primers for human genes | |
| --- | --- |
| Gene | **Primer** |
| CHRNA10 | CTTCCTTCCTAGGTGGCTGC |
| CHRNA10 | ACTCGACTGAGAATCCCCGA |
| CERKL-f | CTGCAGGCCTGTGAACAGAT |
| CERKL-r | AGCTGTGGCCGAATTTTGAAC |
| SMYD1-f | TGCATGAACTCAGGCAGACC |
| SMYD1-r | GTGACGTTCTTGGGTGGTGT |
| FA2H-f | GATGAGGTTCCTGCCCCTTC |
| FA2H-r | ATAGCTCCGCAGCAAACAGT |
| GAPDH-f | TATAAATTGAGCCCGCAGCC |
| GAPDH-r | ATGGTGTCTGAGCGATGTGG |
| RPL30-f | CCGTGAAGAGCTTTGCATTGT |
| RPL30-r | GTTGATCGACTCCAGCGACT |
| Table B. Primers for rat genes | |
| Gene | **Primer** |
| CHRNA10-f | GCTTCTGACCTCACAACCCAC |
| CHRNA10-r | GGGCTTTAGATCCCCCTCCA |
| CERKL-f | AGCGGGTTGTTCTCAGGATG |
| CERKL-r | CCACTGAACACAGACAGGGG |
| SMYD1-f | CTTTGTGCAGGGGTCTCTGG |
| SMYD1-r | CCAAGAACAGTCCCAGCCTC |
| FA2H-f | CTCCTGAAGCCAAGAAGGGG |
| FA2H-r | GCAGTGAGGGCTGTGGTTAT |
| GAPDH-f | CCAGCCCAGCAAGGATACTG |
| GAPDH-r | GGGAGATGCTCAGTGTTGGG |

**Supplementary methods: TOTAL RNA ISOLATION:** A small sample (~100 mg) of frozen BA9 sample (N=31) was taken, and tissue was homogenized in 1 mL of QIAzol lysis buffer reagent (Qiagen, Germantown, MD, USA) using Polytron PT 2100 with a tip PT-DA 07/2EC-D100 (Fisher Scientific) in a 4ºC cold room. From the homogenate, RNA was isolated using RNeasy Lipid Tissue Mini Kit (Qiagen, 74804) and on-column DNase digestion with RNase-Free DNase Set (Qiagen, 79254), as per the manufacturer’s instructions. RNA integrity was assessed by electrophoresis (Agilent 2200 Tape Station system), and only RNA samples with RIN (RNA integrity number) values of ≥6 were subjected to further analyses. RNA samples were shipped to Novogene Corporation Inc (Sacramento, CA, USA) for RNA sequencing. Normalization and differential gene expression analyses were performed using the DESEQ2 R package. Differentially expressed genes were extracted by applying a p-value (<0.05), and base expressed genes were set to a 5-fold change cutoff for the genes. Differentially expressed genes that passed the previously described cutoffs were used to get the biotype classification using Ensembl database REST API, and composition analysis based on biotypes was generated in R. Downstream analyses were built using coding genes
